# Ocean variability and air-sea fluxes produced by atmospheric rivers

**DOI:** 10.1038/s41598-019-38562-2

**Published:** 2019-02-15

**Authors:** Toshiaki Shinoda, Luis Zamudio, Yanjuan Guo, E. Joseph Metzger, Chris W. Fairall

**Affiliations:** 1 Texas A&M University - Corpus Christi, Corpus Christi, USA; 2 Florida State University, Tallahassee, USA; 3 University of California Los Angeles, Los Angeles, USA; 4 Naval Research Laboratory- Stennis Space Center, Mississippi, USA; 5 NOAA/ESRL, Boulder, USA

## Abstract

Atmospheric rivers (ARs) cause heavy precipitation and flooding in the coastal areas of many mid-latitude continents, and thus the atmospheric processes associated with the AR have been intensively studied in recent years. However, AR-associated ocean variability and air-sea fluxes have received little attention because of the lack of high-resolution ocean data until recently. Here we demonstrate that typical ARs can generate strong upper ocean response and substantial air-sea fluxes using a high-resolution (1/12°) ocean reanalysis. AR events observed during the CalWater 2015 field campaign generate large-scale on-shore currents that hit the coast, generating strong narrow northward jets along the west coast of North America, in association with a substantial rise of sea level at the coast. In the open ocean, the AR generates prominent changes of mixed layer depth, especially south of 30°N due to the strong surface winds and air-sea heat fluxes. The prominent cooling of SST is observed only in the vicinity of AR upstream areas primarily due to the large latent heat flux. Using a long-term AR dataset, composite structure and variations of upper ocean and air-sea fluxes are presented, which are consistent with those found in the events during CalWater 2015.

## Introduction

Atmospheric rivers (ARs) are narrow elongated regions of strong water vapor transport which typically extend from the (sub)tropics to the mid-latitude^1,2^, and they are responsible for a majority of total poleward water vapor transport at middle and high latitudes over the globe^3,4^. ARs are often connected to the warm sector of extratropical cyclones, and are associated with strong low-level winds.

When strong ARs make landfall, they have substantial impacts on precipitation, floods and snowpack along the coast of many continents including the west coast of US and northern Europe. ARs also influence inland and tropical/polar areas significantly. Because of such an important role of ARs in the global water cycle as well as societal impacts caused by extreme weather events in many coastal areas, atmospheric processes associated with ARs have been studied intensively in the last one and half decades^5–12^.

Because of the extreme rainfall produced by ARs, previous studies mostly focused on hydrological components such as large moisture transport, heavy precipitation, flooding, and snowpack. Yet a recent study emphasizes the importance of extreme wind events produced by ARs^13^. The analysis of global AR datasets indicates that they are associated with a doubling or more of the typical wind speed compared to all storm conditions, and a 50–100% increase in the wind speed for extreme events^13^. The AR-induced strong winds are often associated with large pressure gradients between an extratropical cyclone and anticyclone located on the eastward and equatorward sides of the cyclone^14^. Given that such strong winds often produce large air-sea fluxes of momentum, heat, and moisture, it is likely that ARs could generate substantial ocean responses. The upper ocean variability produced by ARs could then in turn feedback on the ARs through sea surface temperature (SST) changes and air-sea fluxes. However, ocean response to ARs has not been emphasized in previous studies partly because of the lack of high-resolution upper ocean data which can resolve narrow coastal areas as well as open oceans. This study investigates upper ocean variability and air-sea fluxes produced by ARs using a high-resolution ocean reanalysis along with air-sea flux data sets based on satellite and reanalysis products.

While making landfall ARs have been routinely monitored by observational networks in many locations (especially in North America), but their behavior over the oceans is much less observed until recently. For the purpose of better understanding the evolution and structure of ARs including those over the open ocean, a new campaign, CalWater 2015, was designed and conducted for the period January-March, 2015^15,16^. In addition to a variety of aircraft and ground-based observations, ship-based observations were also conducted, which include cloud and precipitation radar, wind profiler, and measurements of air-sea fluxes, ocean mixed layer structure and currents. These data provide useful information for the model-based analysis for the ocean variability including the model validation.

In this study, we examine the ocean response to ARs such as the evolution of the mixed layer, SST and sea level and the generation of upper ocean currents by surface forcing fields generated by ARs using a high-resolution ocean reanalysis product as well as other data sets based on satellite observations. The ocean variability produced by ARs observed during CalWater 2015 is first described and compared with some of the *in-situ* data collected during the field campaign. Then the composite evolution of ocean variability and air-sea fluxes are examined and compared with the CalWater 2015 case study.

## Data and Methods

### High resolution ocean reanalysis

The ocean reanalysis product used in this study is similar to the US Navy’s operational Global Ocean Nowcast/Forecast System^17^. The system is comprised of the 0.08° HYbrid Coordinate Ocean Model (HYCOM^18^) and the Navy Coupled Ocean Data Assimilation (NCODA^19^). Since the detailed description of HYCOM, NCODA, and data assimilation method are presented in other papers^17,18,20,21^, only aspects relevant to this study is briefly described in the following.

HYCOM uses the hybrid coordinate that is isopycnal in the open, stratified ocean, but smoothly reverts to a terrain-following coordinate in shallow coastal regions, and to z-level coordinates in the mixed layer and/or unstratified seas. The surface fluxes of momentum, heat and freshwater are derived from the 1-hourly Climate Forecast System Reanalysis (CFSR) products provided by National Centers for Environmental Prediction (NCEP)^22^. The data assimilated by NCODA include remotely sensed sea surface height (SSH), sea surface temperature (SST) and sea ice concentration as well as *in-situ* surface and subsurface observations of temperature and salinity. These include synthetic temperature profiles computed using the Improved Synthetic Ocean Profile (ISOP^21^), in which the vertical profile at a given location is constructed by projecting remotely observed SSH and SST downward from the surface using a global database of statistical relationships. It should be noted that the use of ISOP substantially improved the subsurface temperature profile in comparison to the previous version of the reanalysis that uses the Modular Ocean Data Assimilation System (MODAS)^23,24^.

While the HYCOM reanalysis is available for 1993–2015, output for the period of 2011–2015 are used in this study since the surface atmospheric forcing fields are changed from CFSRV1 to Climate Forecast System Version 2 (CFSV2; https://www.ncdc.noaa.gov/data-access/model-data/model-datasets/climate-forecast-system-version2-cfsv2) from 2011 with an accompanying horizontal resolution increase to 0.205° (from 0.3125°). Note that the time period of 5 years (2011–2015), which includes many AR events in the northeast Pacific, is sufficiently long for the present study.

The HYCOM ocean reanalysis has been extensively validated in the last few years^25,26^. Because of the high resolution of the model, it is able to resolve upper ocean structure near the boundary including the narrow boundary currents^27^, which are difficult to monitor by satellite observations only. The HYCOM reanalysis is available at https://hycom.org/dataserver/gofs-3pt1/reanalysis.

### Air-sea fluxes and surface atmospheric variables

Latent and sensible heat fluxes and evaporation derived from the Objectively Analyzed air-sea Fluxes (OAFlux) products^28,29^ are used to describe air-sea fluxes associated with ARs. SST, specific humidity at 2 m height, and wind speed at 10 m height, that are used for the air-sea flux calculation, are also used for the analysis. In addition, daily mean winds at 10 m from CFSV2, that are used for the HYCOM reanalysis, are analyzed for describing the near-surface wind vector. Surface shortwave and longwave radiation is obtained from the Clouds and the Earth’s Radiant Energy System (CERES^30^).

### *In-situ* data

CalWater 2015 was conducted during January-March 2015^15^. In this study, upper ocean currents measured by shipboard acoustic doppler current profiler (ADCP) and air-sea fluxes calculated using meteorological variables measured on board are used. During the field campaign, R/V Ron Brown stayed at 37°N, 127.2°W when strong AR events passed through this location. ADCP and surface flux data are primarily used for the validation of the HYCOM reanalysis and OAFlux data. Surface fluxes and ADCP data collected during CalWater 2015 are available at ftp://ftp1.esrl.noaa.gov/psd3/cruises/CalWater2015.

Daily mean sea level data measured by tide gauges from the two stations, Neah Bay, Washington 48°22′N, 124°37′W and South Beach, Oregon 44°38′N, 123°03′W, located at the west coast of North America are used to monitor sea level variation associated with ARs. The data are obtained from the University of Hawaii Sea Level Center.

### Compositing method

Guo *et al*.^14^ constructed composites of atmospheric variables associated with ARs using the long-term global AR data set created by Guan and Waliser^31^. Here a similar method is used to construct composites of ocean variability and air-sea fluxes associated with ARs. ARs are identified based on the characteristics of the integrated water vapor transport (IVT) at 6 hourly intervals^31^. The AR data set includes the time and location of the AR centroid and IVT at the AR center. The AR events detected in the northeast Pacific domain, 15°N-60°N, 180°E-100°W, are used for the analysis. Then daily variables relevant to upper ocean variability and air-sea fluxes are composited over a 60° longitude by 40° latitude domain centered at the centroid of ARs, using AR events for the 5-year period of 2011–2015. Since the AR data are 6 hourly, ARs are often identified multiple times within a day. In this case, the same daily values are used multiple times for the composite. To exclude weak events, only AR events in which IVT at the center exceeds 500 kg m^−1^ s^−1^ are used.

## Results

### Lanfalling AR events during CalWater 2015

**Figure 1:**
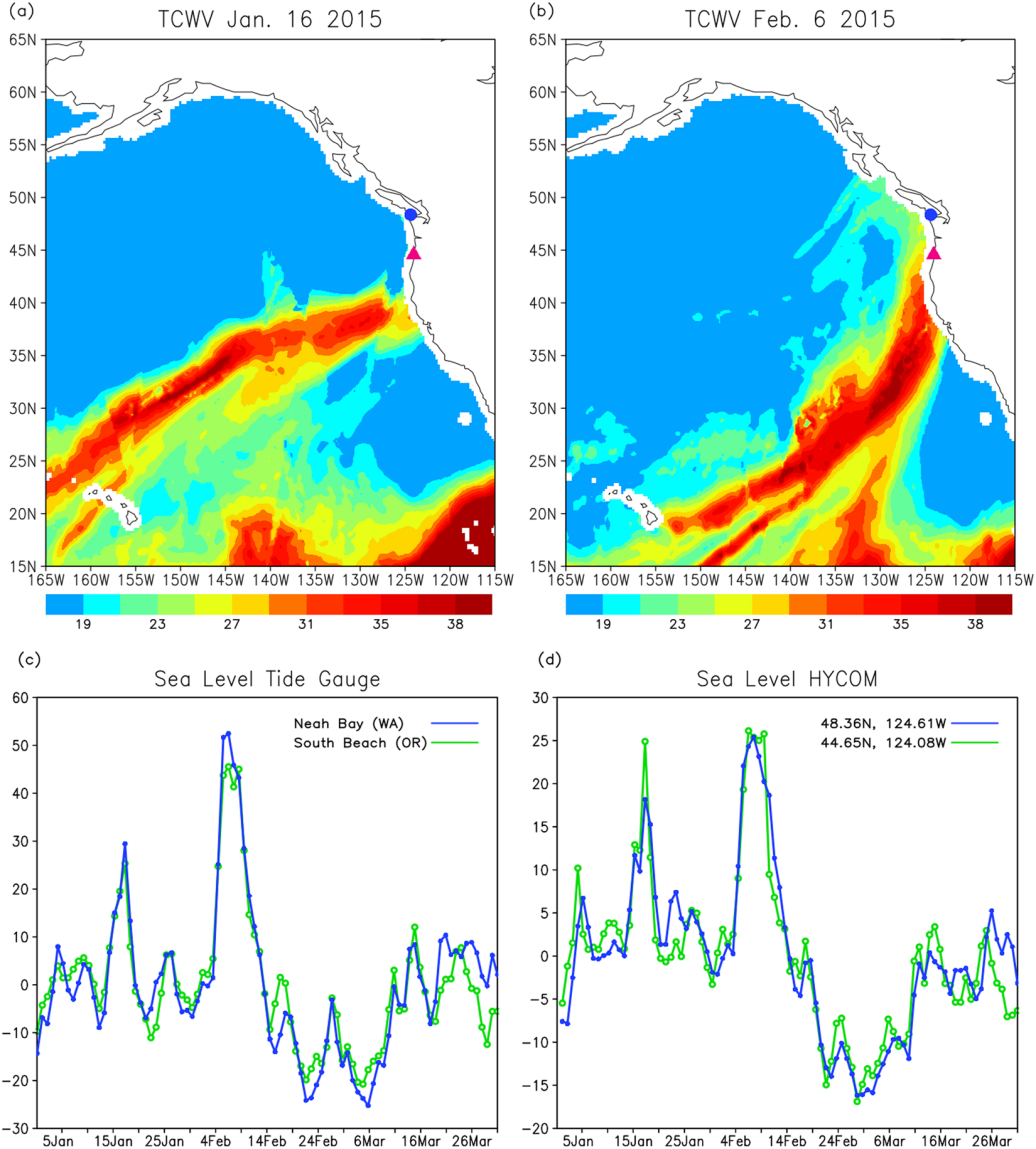
Total column integrated water vapor (mm) on (**a**) January 16, and (**b**) February 6, 2015 derived from Special Sensor Microwave Imager (SSMI) data. The circle (triangle) mark indicates the location of sea level station Neah Bay (South Beach). (**c**) Time series of sea level (cm) from the tide gauge stations Neah Bay 48°22′N, 124°27′W (blue line) and South Beach 44°38′N, 124°03′W (green line). (**d**) Time series of sea level from the HYCOM reanalysis at the grid point closest to the Neah Bay station 48°22′N, 124°38′W (blue line) and the South Beach station 44°39′N, 124°05′W (green line). Note that the scale of vertical axis in (**c**) and (**d**) are different.

Two strong landfalling AR events were observed during CalWater 2015 (Fig. 1). The first event occurred in mid-January, with the landfall on the west coast of North America around January 16 (Fig. 1a). The second event was observed early February in the northeast Pacific, and it made landfall around February 6 (Fig. 1b). The upper ocean response to the event during early February, in which the ocean variability was measured by ship observations, is first discussed in the following sections.

#### Sea level, upper ocean currents, and mixed layer depth

A prominent sea level rise is evident during the two landfalling AR events in mid-January and early February (Fig. 1c). In particular, the magnitude of the sea level rise during the event in early February exceeds 50 cm, and the duration of the significant sea level rise is about 5–7 days. Such a large sea level rise was not observed in other periods of the entire winter (January to March) in 2015.

The sea level rise associated with AR events is also evident in the high-resolution ocean (HYCOM) reanalysis (Fig. 1d). The time series of tide gauge data and HYCOM reanalysis are very similar (correlation coefficient: 0.91 for South Beach and 0.89 for Neah Bay), and two distinct peaks of sea level rise are found in both time series. This suggests that the reanalysis is able to capture oceanic processes that cause the prominent sea level rise generated by ARs. Note that the magnitude of the sea level rise in the HYCOM reanalysis is smaller, partly because the variation in the reanalysis is on the scale of the model grid (~6 km resolution at these latitudes). Nevertheless, the good agreement of two time series and the presence of prominent sea level rise during AR events indicate that the reanalysis provides a useful tool to investigate the coastal ocean response to surface forcing fields produced by ARs and its relation to the open ocean variability.

**Figure 2:**
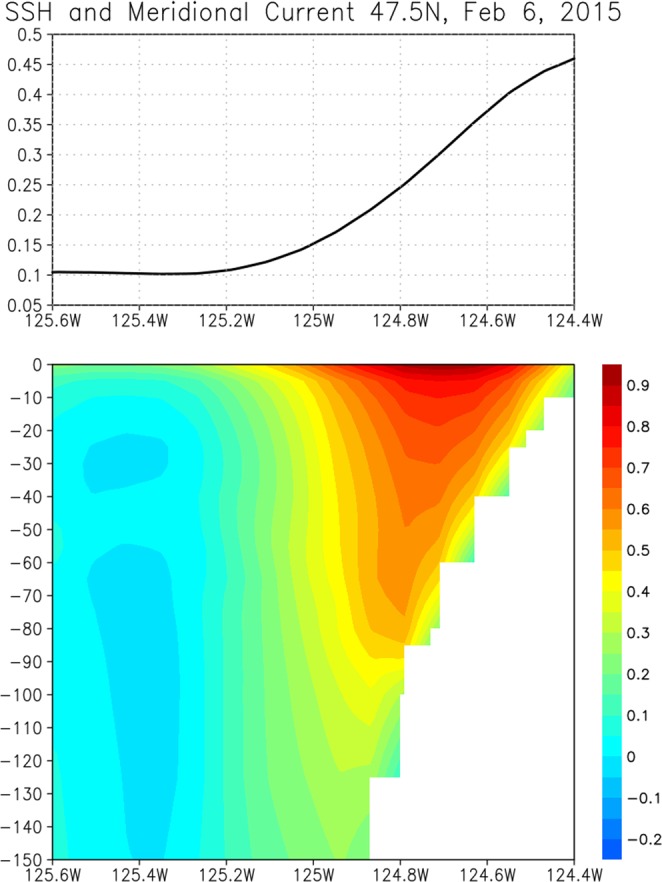
Upper panel: Sea surface height (m) along 47.5°N near the coast on February 6, 2015. Lower panel: Meridional velocity (m/s) along 47.5°N near the coast. Both are from the HYCOM reanalysis.

In association with sea level rise near the coast, a strong northward jet with the maximum velocity of about 1 m/s is generated (Fig. 2). The current width is about 0.5° (38 km), and the vertical extent is about 100 m. Although the strength of the coastal currents varies with locations, the strong coastal jets are generated in the large areas along the coast of North America (Supplementary Fig. S1).

**Figure 3:**
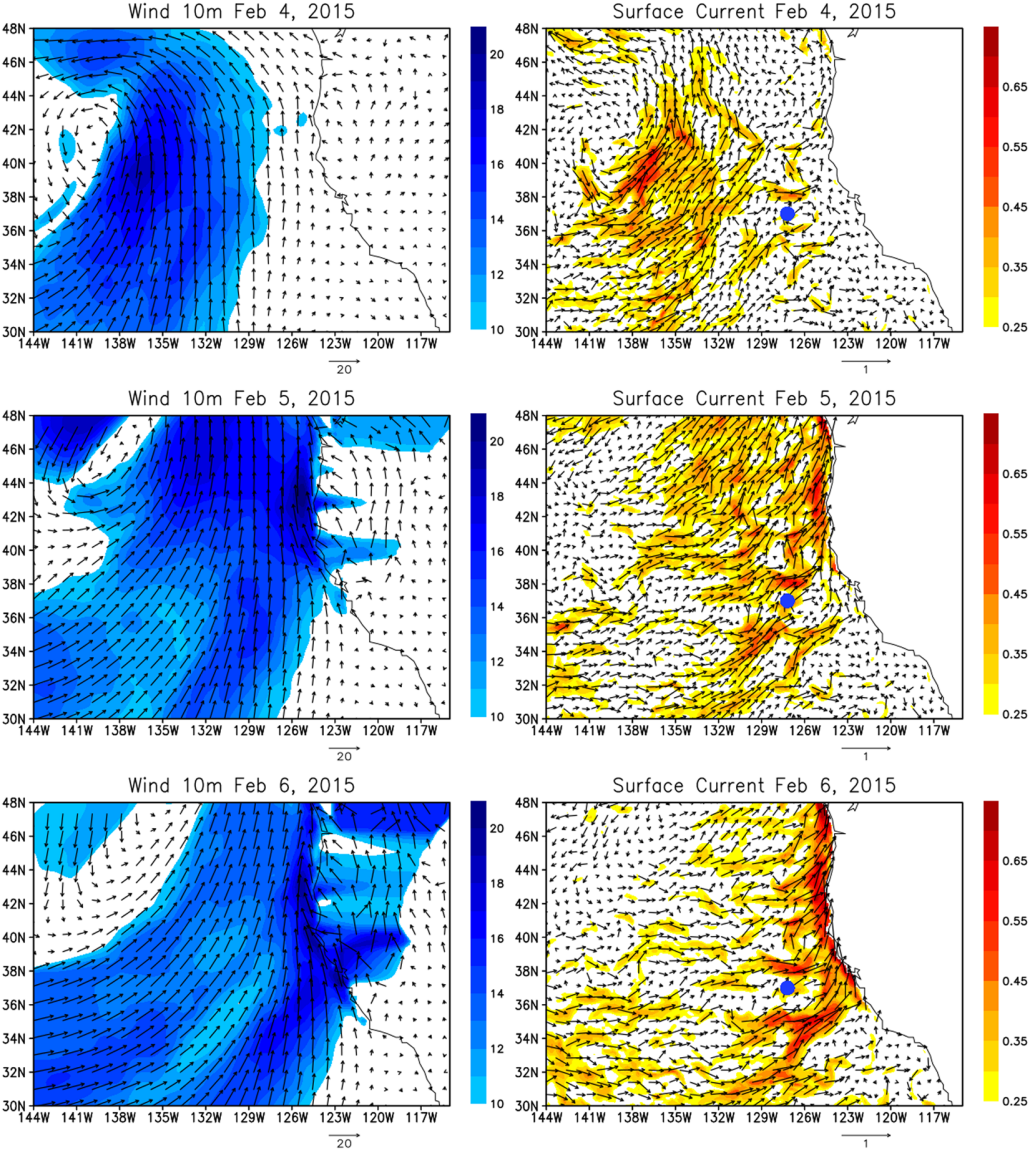
Left panels: Winds at 10 m height on February 4 (upper panel), February 5 (middle panel), and February 6 (lower panel), 2015 from CFSV2. Shading indicates wind speed (m/s). Right panels: Surface currents on February 4 (upper panel), February 5 (middle panel), and February 6 (lower panel), 2015 from the HYCOM reanalysis. Shading indicates current speed (m/s). The blue mark indicates 37°N, 127.2°W where the measurements are conducted by the R/V Ron Brown in early February.

Since the HYCOM reanalysis can be used to describe the large-scale ocean variability, it is further investigated how the sea level rise and strong coastal jet are connected to the open ocean circulation. Figure 3 shows winds at 10 m and surface currents during the period when the AR is making landfall in early February. As in most AR events in the northeast Pacific, a cyclonic circulation and strong southwesterlies on the southeast side of the extratropical cyclone center are evident. Such relatively narrow and strong surface winds are the result of a large pressure gradient between extratropical cyclone and anticyclone, which is evident in most ARs^14^. As the AR makes landfall on February 6, the strong southwesterlies reach the west coast of North America. The response of ocean surface currents to these surface winds is detected in the high-resolution (the eddy-resolving) HYCOM ocean reanalysis. Despite the noisy fields of surface currents due to active mesoscale eddies, eastward movement of the areas of strong zonal current associated with the strong southwesterlies are clearly found. When the AR makes landfall, strong currents against the west coast of North America hit the boundary, generating strong northward along-shore currents (Fig. 3, right panels).

The acceleration of zonal currents produced by the AR event was observed during the CalWater 2015 field campaign (Supplementary Fig. S2). During early February, R/V Ron Brown stayed at 37°N, 127.2°W (blue dot in Fig. 3) when the AR-induced strong southwesterlies passed over the location. The HYCOM reanalysis indicates that strong zonal currents are mostly generated in the upper 50 m at this location, and currents below 50 m are much weaker. Yet the significant acceleration is evident below 50 m. Although the shipboard ADCP used during CalWater 2015 measured currents only below around 60 m depth, the acceleration of the zonal currents is clearly evident. The timing and magnitude of the zonal current acceleration of the HYCOM analysis is consistent with the ADCP observations.

**Figure 4:**
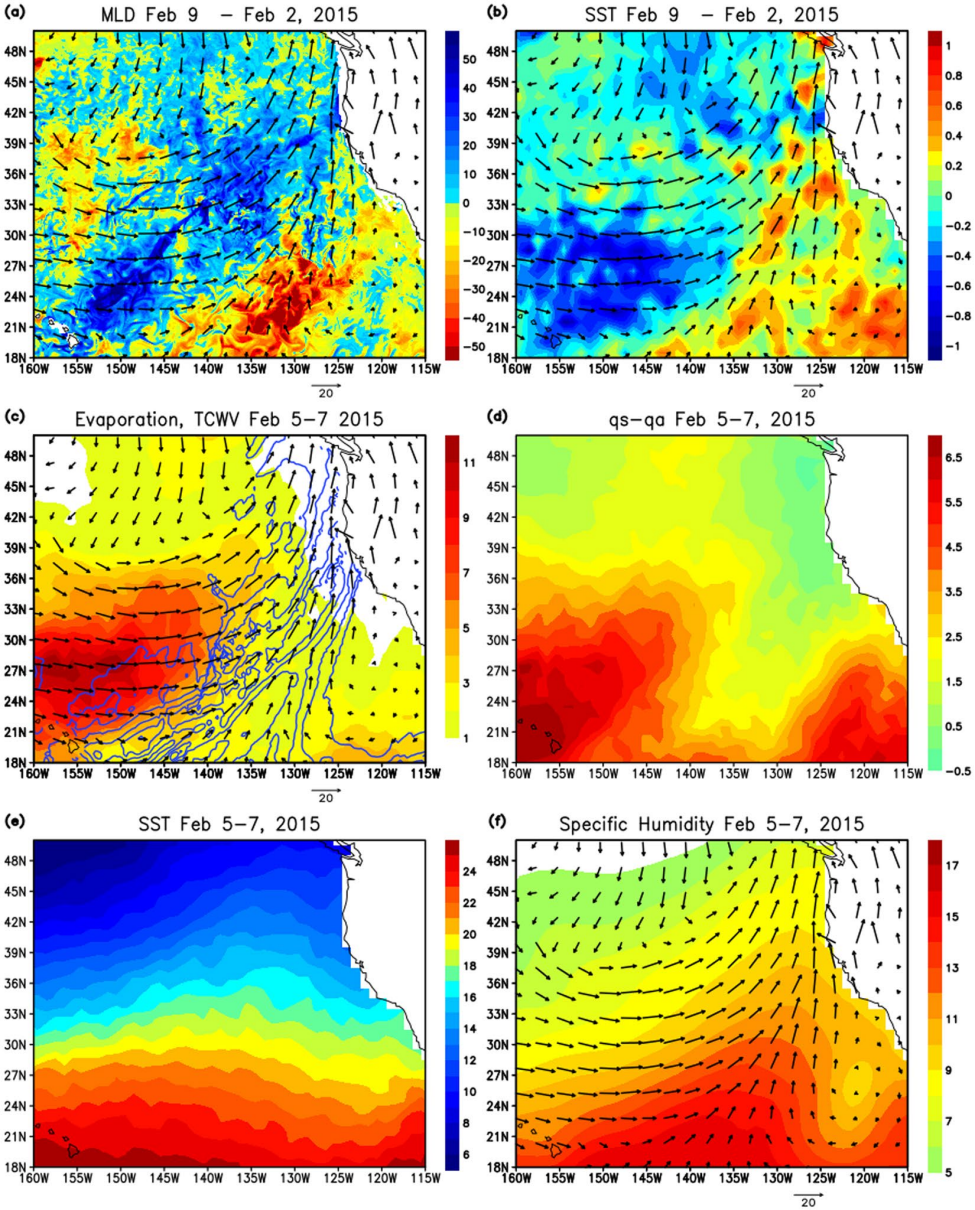
(**a**) The difference of mixed layer depth (m: shading) between the periods before the AR event (February 1–3, 2015) and after the event (February 8–10, 2015), and winds (m/s) at 10 m (arrows) on February 6, 2015. (**b**) Same as (**a**) except the shading indicates SST (°C) from OAFlux. (**c**) Evaporation (mm/day: shading) on February 5–7, 2015, winds (m/s) at 10 m (arrows) on February 6, and total column integrated water vapor (contour) on February 6. The contour starts from 20 mm and the interval is 4 mm. (**d**) Saturation specific humidity at the sea surface (q_s_) minus specific humidity at 2 m (q_a_) from OAFlux (**e**) SST on February 5–7, 2015 from OAFlux (**f**) Specific humidity at 2 m (g/kg: shading) on February 5–7, 2015, and winds (m/s) at 10 m (arrows) on February 6.

Figure 4a shows the difference in mixed layer depth (MLD) between before and after the AR landfall. A substantial deepening of the mixed layer is found in the areas of strong southwesterlies associated with the AR, suggesting that the deepening is generated primarily by strong wind forcing. However, the deepening is more prominent in the upstream region south of 30°N, although the wind is stronger in the downstream areas. This can be explained by the difference in surface heat fluxes between upstream and downstream regions, which will be described in the following section.

#### Air-sea fluxes and SST

While OAFlux data are validated in many locations and time periods, they are also compared with those estimated from the data collected during CalWater 2015. The latent heat flux from OAFlux is consistent with that estimated from CalWater 2015 observations (Supplementary Fig. S3). The good agreement suggests that the OAFlux data are suitable for describing spatial and temporal variations of latent heat fluxes and evaporation generated by ARs.

**Figure 5:**
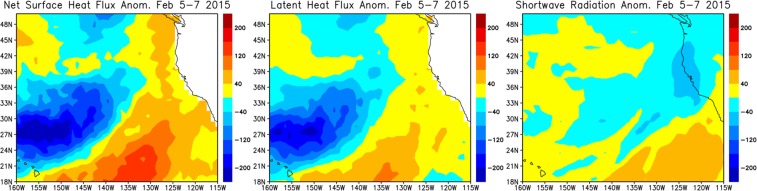
Left panel: Net surface heat flux anomaly (W m^−2^) relative to climatology on February 5–7 from OAFlux and CERES. Positive values indicate warming of the ocean. The climatology is calculated for the period 2001–2015. Middle panel: Same as the left panel except for latent heat flux anomaly. Right panel: Same as the left panel except for surface shortwave radiation anomaly.

Figure 4b shows the difference in SST between before and after the AR landfall. A large SST cooling is found only in the vicinity (northwest side) of AR upstream areas. This spatial pattern is very similar to the evaporation (latent heat flux) (Fig. 4c). The magnitude of the cooling in other components of flux anomaly, including shortwave radiation, is much smaller (Fig. 5), suggesting that the large cooling in the vicinity of AR upstream areas is primarily caused by the evaporative cooling. The spatial pattern of evaporation, total column integrated water vapor (TCWV), and surface winds (Fig. 4c) also suggests the potentially important role of evaporation in the moisture budget associated with ARs. The strong zonal winds around the area of maximum evaporation north of Hawaii could bring the surface moisture towards the area of large TCWV, and thus the surface moisture derived from the AR-induced evaporation could enhance the TCWV in the warm sector of extratropical cyclone.

The evaporation in the downstream areas is very small or close to zero, despite that the strong surface winds are observed. This is explained by the difference in the vertical humidity gradient near the surface between the upstream and downstream areas. The difference between saturation specific humidity at the surface (*q*_*s*_) and specific humidity at 2 m (*q*_*a*_) is shown in Fig. 4d. The spatial pattern of *q*_*s*_*-q*_*a*_ is quite similar to that of surface evaporation. *q*_*s*_*-q*_*a*_ in the AR downstream areas are close to zero or even negative, which results in very small evaporation even though winds are strong. Such small values of *q*_*s*_*-q*_*a*_ is due to a combination of cold SST (Fig. 4e) and the increase of *q*_*a*_ caused by enhanced moisture advection from the tropics (Fig. 4f).

In addition to the large cooling on the northwest side of the AR upstream areas, the significant SST warming is found on the east side of the cooling area. The warming is primarily generated by the anomalous surface heat fluxes (Fig. 5). The positive surface heat flux anomaly (warming) is primarily due to the reduction of evaporative cooling. As discussed above, the warming areas are located around the large moisture transport by the AR-induced surface winds and thus the vertical humidity gradient is very small even though the wind is relatively strong, resulting in the reduction of evaporative cooling. The enhanced shortwave radiation also contributes to the warming (Fig. 5). It should be noted that the net surface heat flux during this period derived from the HYCOM reanalysis is similar to that calculated from OAFlux and CERES.

During the event, the deepening of the mixed layer is found in the area of SST cooling while the mixed layer becomes shallow in the area of warming (Supplementary Fig. S4). This suggests that the warming is enhanced by the shallowing of the mixed layer while the deeper mixed layer tends to reduce the cooling produced by the anomalous surface heat flux. The vertical temperature structure during the event shows that the surface cooling extends to around 70–80 m (Supplementary Fig. S5). While such mixed layer evolution suggests the contribution of the entrainment cooling, the upper ocean heat budget analysis is necessary to further quantify the relative importance of each process.

The same analysis described above has been conducted for the AR event during mid-January (Supplementary Figs S6, S7). Although the spatial structure of the January event is different, major features found during the event in early February are quite similar. These include the generation of northward jets that are connected to the surface currents toward the coast, enhanced SST cooling in the vicinity of the AR upstream areas produced by evaporative cooling, and significant deepening of the mixed layer.

### Composite analysis

Composites of ocean variability and air-sea fluxes associated with ARs are constructed to demonstrate that many of the processes identified in the AR events observed during CalWater 2015 are common in most other events. While the compositing technique used in this study is simple as explained in the data and method section, such composites, including those for atmospheric variables, have not been created until recently because of the lack of long-term objectively detected AR data base^14^. Following the compositing method described in the data and method section, composites are formed using 1584 AR events in the northeast Pacific during 2011–2015. Note that in this study an AR event is the “snapshot” AR at each 6 hours, which is different from more traditional definition of an AR event that covers the entire lifecycle.

**Figure 6:**
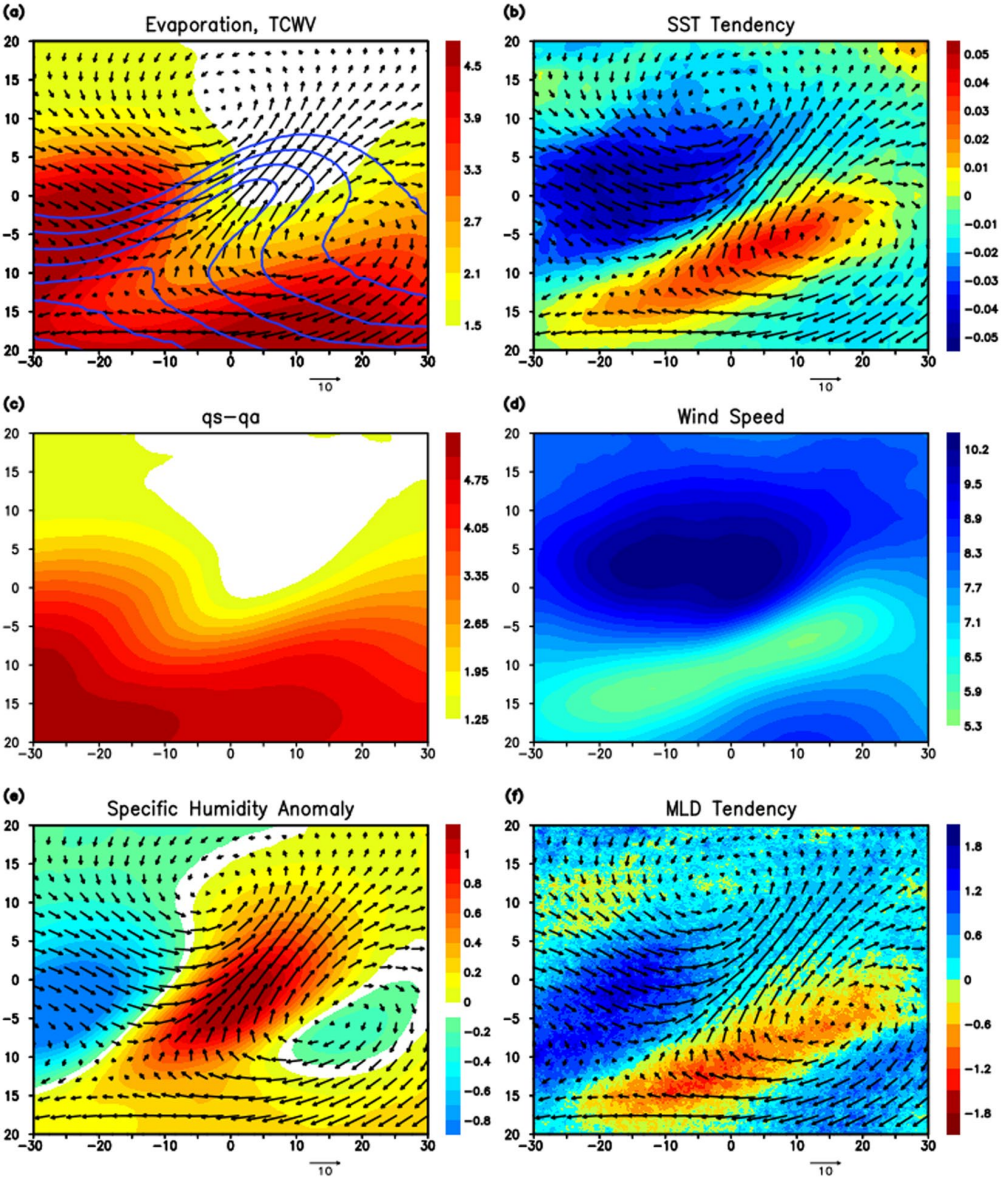
(**a**) Composite of evaporation (mm/day: shading), TCWV (mm: contour), and winds at 10 m (m/s: arrows) for the period 2011–2015. The contour starts from 21 mm, and the interval is 3 mm. Horizontal (vertical) axis is longitude (latitude), in which 0° longitude, 0° latitude are defined as the location of the AR center. See text for the detail of the compositing method. (**b**) Composite of SST tendency (C/day: shading) from OAFlux and winds at 10 m (m/s: arrows) from the CFSV2 reanalysis. SST tendency is calculated as the difference between the 2-day average SST before and after the AR event. (**c**) Composite of saturation specific humidity at the surface minus specific humidity (g/kg) at 2 m. (**d**) Composite wind speed used to calculate the evaporation. (**e**) Composite of specific humidity anomaly at 2 m (shading) relative to climatology and winds at 10 m (m/s: arrows). (**f**) Composite of mixed layer depth tendency (m/day: shading) and winds at 10 m (m/s: arrows).

The spatial pattern of composite evaporation, winds, and TCWV associated with ARs (Fig. 6a) are similar to those identified in the event during CalWater 2015 (Fig. 4c). The maximum evaporation is located in the vicinity of AR upstream. The low-level moisture produced by the large evaporation could be advected toward the area of high TCWV by strong surface zonal winds. The composite of SST tendency shows the maximum cooling in the area near the AR upstream (Fig. 6b), which is also similar to the CalWater 2015 case (Fig. 4b).

Very small evaporation in the downstream areas is explained by the small *q*_*s*_*-q*_*a*_ (Fig. 6c) although the surface winds are strong (Fig. 6d). The small *q*_*s*_*-q*_*a*_ in the downstream area is due to the cold SST and enhanced surface specific humidity. The advection of low level moisture significantly contributes to the low *q*_*s*_*-q*_*a*_ in the downstream area (Fig. 6e). The spatial distribution of composite MLD tendency is similar to that in the CalWater 2015 case, although the difference of ML deepening between upstream and downstream areas appears to be more prominent in the composite.

**Figure 7:**
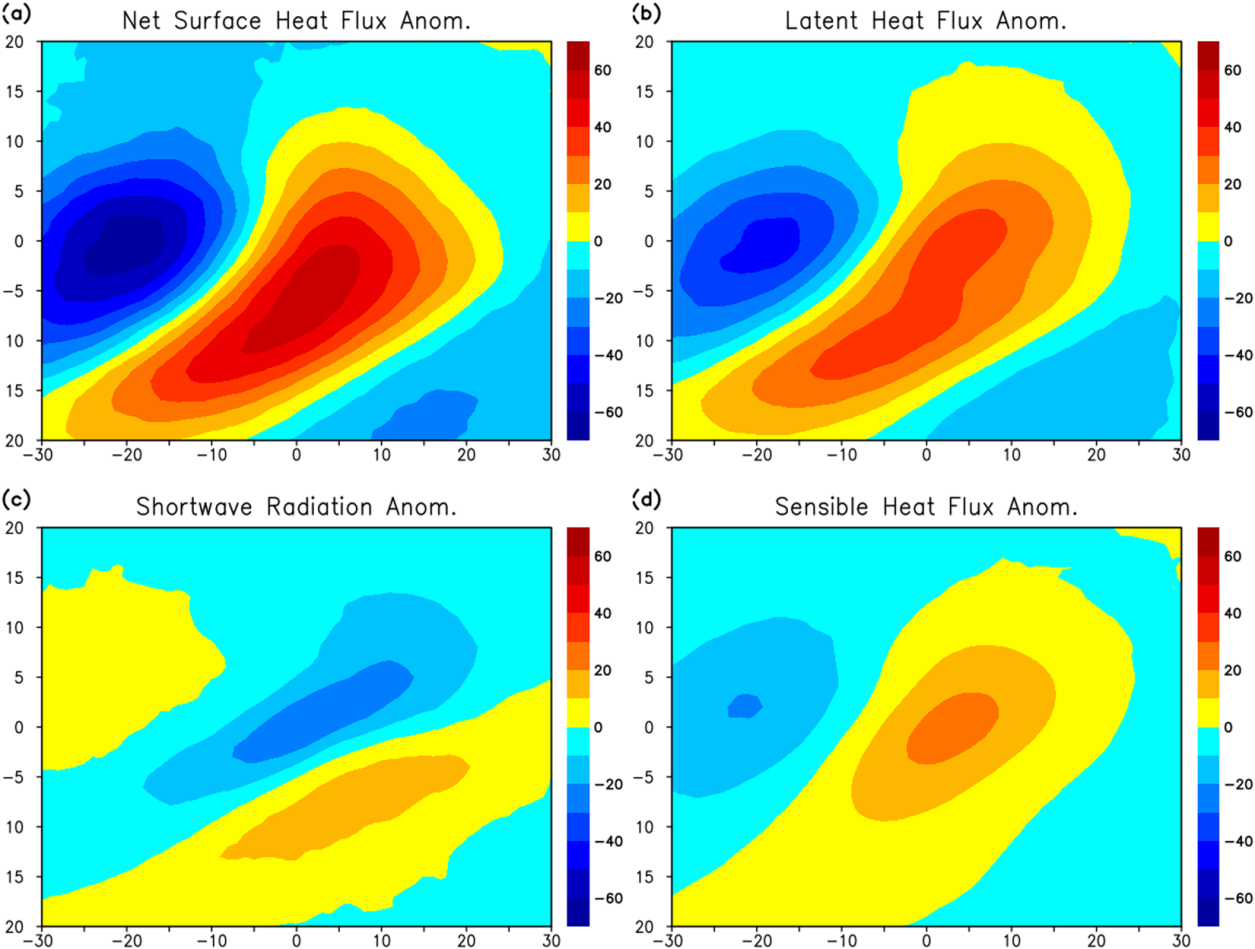
(**a**) Composite of net surface heat flux (W m^−2^) anomaly from OAFlux and CERES. Positive values indicate the warming of the ocean. (**b**) Same as (**a**) except for latent heat flux anomaly. (**c**) Same for (**a**) except for shortwave radiation anomaly. (**d**) Same as (**a**) except for sensible heat flux anomaly.

The spatial pattern of SST tendency shows a dipole-like structure in which the strong cooling is associated with the warming in the east and southeast side of the AR center. The warming is generated by the anomalous surface heat flux, which shows the spatial pattern similar to the SST tendency (Fig. 7a). The surface warming is primarily caused by the anomalous latent heat flux as the evaporative cooling is largely reduced by the moisture transport (Fig. 7b). The anomalous shortwave radiation and sensible heat flux significantly contribute to the warming (Fig. 7c,d).

**Figure 8:**
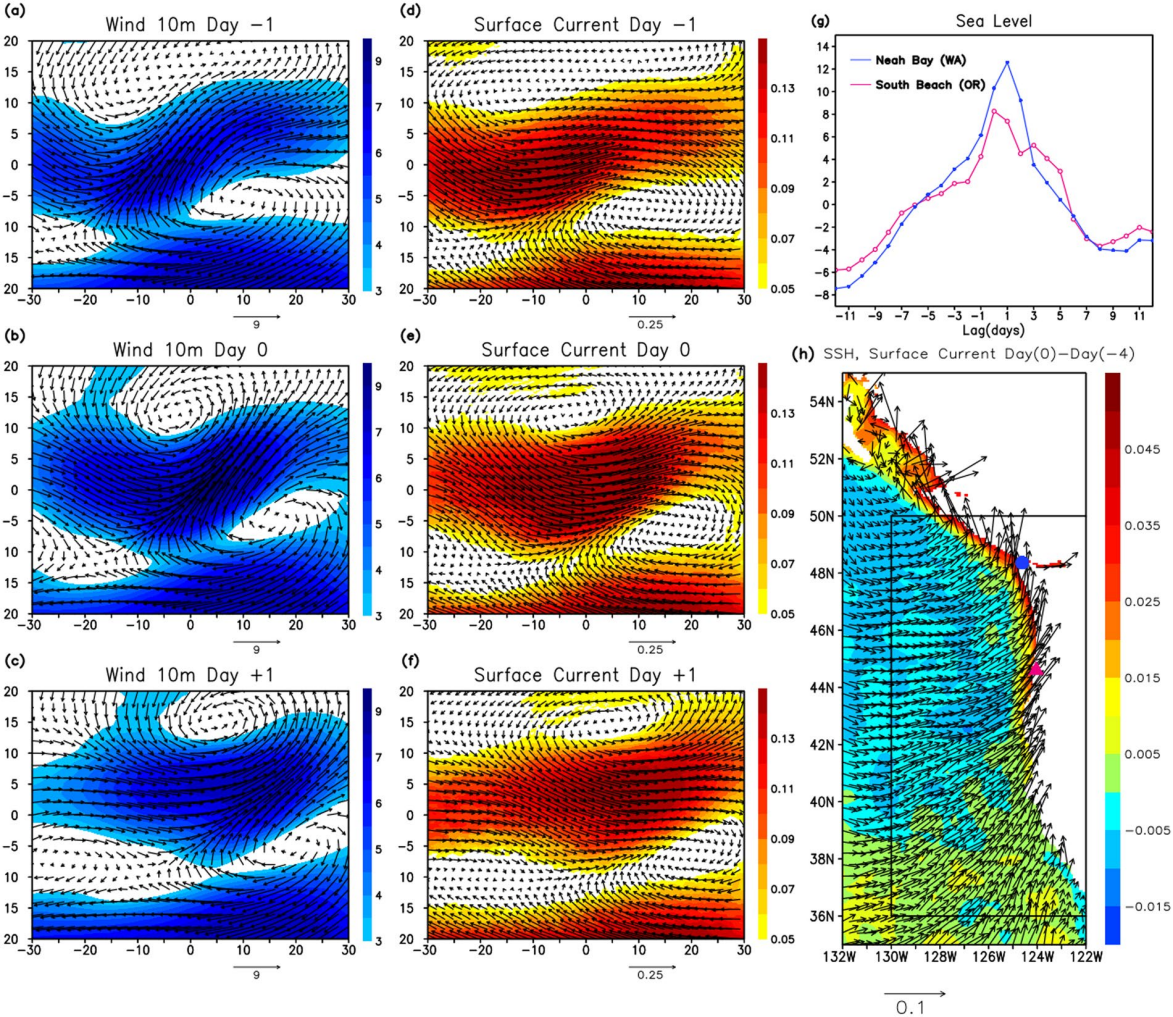
(**a**–**c**) Composite of wind vector (arrows) and wind speed (m/s: shading) on Day −1 (**a**), Day 0 (**b**), and Day +1 (**c**). Horizontal (vertical) axis is longitude (latitude), in which 0° longitude, 0° latitude are defined as the location of the AR center on Day 0. (**d**–**f)** Same as (**a**–**c**) except for surface currents (m/s) from the HYCOM reanalysis. The method for constructing composite is the same as in Fig. 6. (**g**) Composite of sea level (cm) variation at the tide gauge stations Neah Bay 48°22′N, 124°27′W (blue line) and South Beach 44°38′N, 124°03′W (magenta line). The composite is formed for AR events that entered in the box in (**h**) and made landfall. (**h**) The difference of composite sea surface height (m: shading) and surface currents (m/s: arrows) between Day 0 and Day −4 from the HYCOM reanalysis.

The composite evolution of surface currents and winds is similar to those in the event during CalWater 2015 (Fig. 8a–f). The enhancement of strong zonal currents is produced by southwesterly winds. These winds near the coastline of North America could generate prominent sea level rise and strong northward currents along the coast, which is further demonstrated by the different composite that uses only the AR events observed near the coast. Figure 8g shows the composite of tide gauge sea level data using AR events in which the AR centroid entered in the area near the coast 36°N–50°N, 130°W–122°W (shown as the box in Fig. 8h) and made landfall. The significant sea level rise produced by the ARs is clearly detected in the composite. The same compositing method is applied to the surface current and SSH from the HYCOM reanalysis (Fig. 8h). The strong currents toward the coast generates sea level rise in the large areas along the west coast, which is associated with narrow strong northward jets.

ARs in the northeast Pacific are generally associated with an anticyclone on the southeast side. Compared to the wind fields produced by a cyclone only, the strong geostrophic winds generated between extratropical cyclone and anticyclone result in producing strong currents toward the north American coast (often nearly perpendicular to the coast line), and thus effectively generating the rapid sea level rise.

In summary, the results of the composite analysis shown in Figs 6–8 are all consistent with the case study for the AR events during the CalWater 2015, suggesting that many of the features identified during the events in CalWater 2015 are found in most other AR events in the northeast Pacific.

## Summary and Discussion

Ocean variability and air-sea fluxes produced by ARs are investigated using the high-resolution (1/12°) ocean reanalysis and other reanalysis and air-sea flux products. During the recent field campaign: CalWater 2015, two strong ARs made landfall along the coast of North America. In this study, ocean variability and air-sea fluxes generated by the AR events during CalWater 2015 are examined first and they are compared with the composite evolution based on a long record of AR data set. The upper ocean and air-sea fluxes observed during the CalWater 2015 are consistent with those from the high-resolution ocean reanalysis and air-sea flux product. The analysis demonstrates that ARs can generate a strong upper ocean response and substantial air-sea fluxes.

AR events observed during the CalWater 2015 field campaign produce large-scale on-shore currents toward the west coast of North America, generating strong narrow northward jets along the coast, in association with a substantial rise of sea level at the coast. In the open ocean in the northeast Pacific, prominent changes of mixed layer depth are evident, especially south of 30°N due to the strong surface winds and air-sea heat fluxes. In contrast, the significant cooling of SST is observed only in the vicinity (northwest side) of AR upstream areas primarily due to the large latent heat flux. The difference in evaporation between downstream and upstream areas is primarily attributed to the difference in vertical humidity gradient near the surface. The vertical humidity gradient is very small or close to zero in the downstream area because of a combination of cold SST and advection of near surface moisture by AR-induced winds.

Using a long-term AR data set, composite structure and variations of ocean and air-sea fluxes associated with ARs are presented. The results indicate that the composite structure and evolution are all consistent with those found in the case study for the events during CalWater 2015. This suggests that oceanic processes and air-sea fluxes associated with ARs identified during the CalWater 2015 are commonly found in most AR events.

While this study mostly focuses on upper ocean variability and the role of air-sea fluxes in affecting the ocean variability, the results also suggest the importance of air-sea fluxes for the atmospheric processes. The comparison of the spatial distribution of evaporation, surface winds, and TCWV indicates that the strong winds blow from the region of large evaporation to the area of a large amount of moisture associated with ARs. This suggests that the advection of moisture by AR-induced strong surface winds provides a significant amount of evaporated water vapor to the center of the AR. Accordingly, the moisture budget associated with ARs may substantially vary from the AR upstream to downstream regions. Hence moisture budget calculations in different areas of ARs are necessary for fully establishing the hydrological cycle associated with ARs.

The comparison of the spatial pattern of latent heat flux and SST tendency suggests that the substantial SST cooling in the vicinity of AR upstream areas are primarily generated by evaporative cooling produced by AR-induced surface winds. However, there are some differences in these spatial patterns. For example, the SST tendency near the AR center is negative (cooling) and the small negative values are evident all the way to the AR downstream areas in the composite, while the latent heat flux anomaly in these areas is close to zero or positive. This suggests that the entrainment cooling produced by the deepening of the mixed layer may contribute to the SST cooling during some of the AR events. The complete upper ocean heat budget calculations using non-assimilative models are necessary to further quantify processes that control SSTs.

Because of the presence of an anticyclone on the southeast side of the AR center, AR-induced winds generate significant wind stress curl and divergence in the broad areas around AR (Supplementary Fig. S8), which are comparable to those around the center of a cyclone. A previous study on the ocean response to explosive cyclones demonstrates that such wind fields could generate significant vertical motion and internal waves in the deep ocean (2000 m–6000 m)^32^. Given such broad areas of anomalous wind stress curl and divergence associated with ARs, they may potentially cause significant mixing in the deep area through generating internal waves. Further study which focuses on the dynamical response to AR wind fields is thus desirable for examining the AR’s impact on the longer time scale climate variability.

The observed sea level rise produced by ARs during CalWater 2015 is substantial (~50 cm). ARs in the northeast Pacific are generally associated with relatively narrow but very strong southwesterlies that effectively generate strong surface currents against the west coast of North America, which results in the rapid increase of sea level. Strong southwesterlies around the AR center are accompanied by the large pressure gradient between the extratropical cyclone and anticyclone located southeast of the cyclone^14^. Such substantial sea level rise could influence longer time scale sea level variation. Although the significant sea level rise generated by each AR occurs for only 5–7 days, the seasonal (3 months) average (January-March) of sea level at Neah Bay in 2015 is about 6 cm lower if the AR-induced rise is excluded. Such a difference is significant for longer term variations, and thus the long-term changes in frequency of ARs could impact the long-term sea level variation along the coast. Accordingly, the results suggest that it may be necessary to consider the influence of short-time scale atmospheric variability such as ARs for identifying the processes that control the long-term sea level variation along the coast.

## Supplementary information

Supplementary Figures
