# Supplementary material for: Ocean variability and air-sea fluxes produced by atmospheric rivers
Source: Sci Rep. 2019 Feb 15;9:2152. doi: 10.1038/s41598-019-38562-2 (PMC6377629; doi:10.1038/s41598-019-38562-2)
Supplement: Supplementary file 1 — Supplementary Figures [file 41598_2019_38562_MOESM1_ESM.docx]

**Supplementary Information for**

**Ocean variability and air-sea fluxes produced by atmospheric rivers**

Toshiaki Shinoda^1^, Luis Zamudio^2^, Yanjuan Guo^1,3^, E. Joseph Metzger^4^, Chris Fairall^5^

^1^Texas A&M University, Corpus Christi
^2^Florida State University

^3^University of California Los Angeles

^4^Naval Research Laboratory- Stennis Space Center

^5^NOAA/ESRL

**Supplementary Figures**


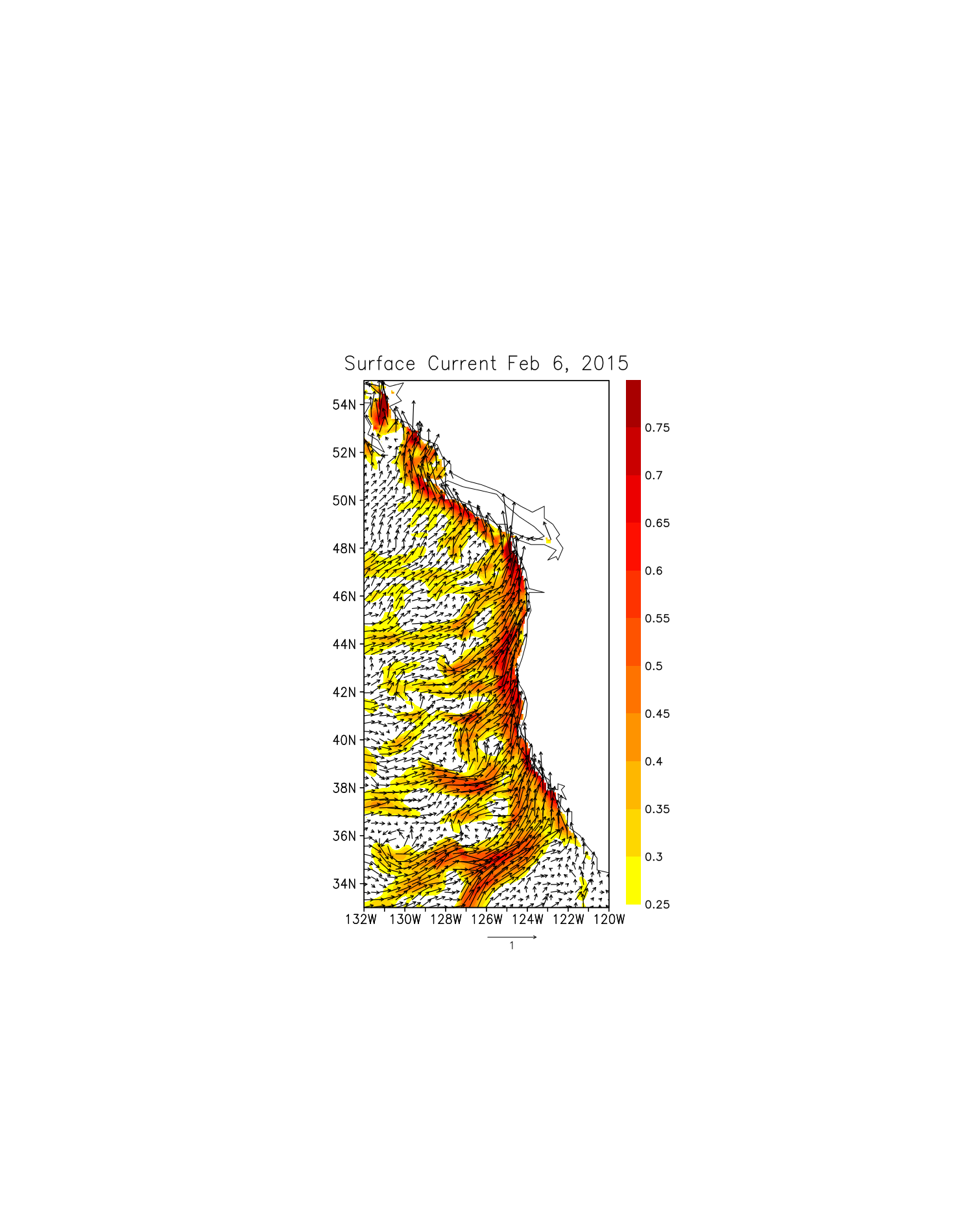


**Figure S1**. Surface currents on February 6, 2015 from the HYCOM reanalysis. Shading indicates the current speed (m/s).


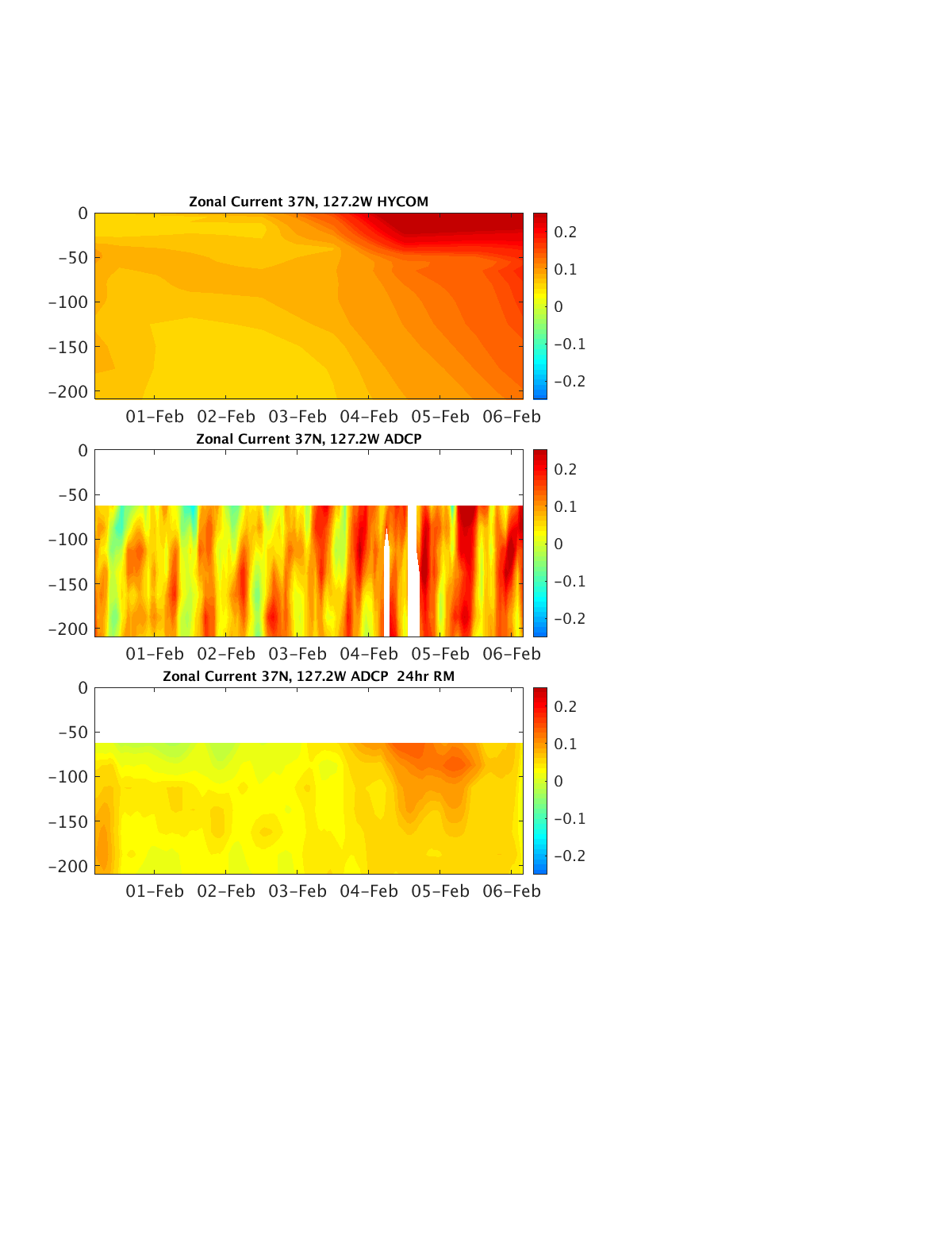


**Figure S2**. Upper panel: Zonal velocity (m/s) at 37.00°N, 127.20°W from the HYCOM reanalysis during February 1-6, 2015. Middle panel: Same as the upper panel except from the ADCP measurements. Bottom panel: Same as the middle panel except the 24 hour running mean is applied to the time series.


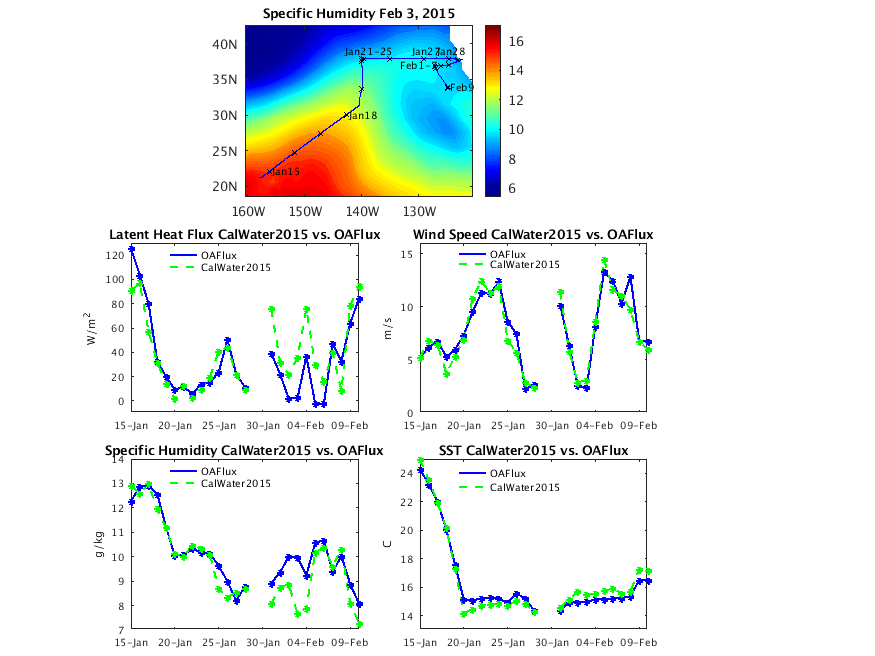


**Figure S3**. Comparison of surface flux and variables from OAFlux and CalWater 2015 observations. Top panel: The cruise track of R/V Ron Brown, and specific humidity (g/kg) at 2 m (shading) on February 3, 2015. Middle left panel: Daily mean latent heat flux (W/m^2^) from OAFlux (blue) and CalWater 2015 (green). The correlation coefficient of two time series is 0.83. Middle right panel: Same as the middle left panel except for wind speed at 10 m (m/s). Lower left panel: Same as the middle left panel except for specific humidity (g/kg) at 2m. Lower right panel: Same as middle left panel except for SST (°C). A significant discrepancy is found in latent heat flux in early February primarily caused by the specific humidity difference. During this period, the observational sites are located around the areas where the horizontal gradient of specific humidity is large (top panel), and thus such a discrepancy is expected.


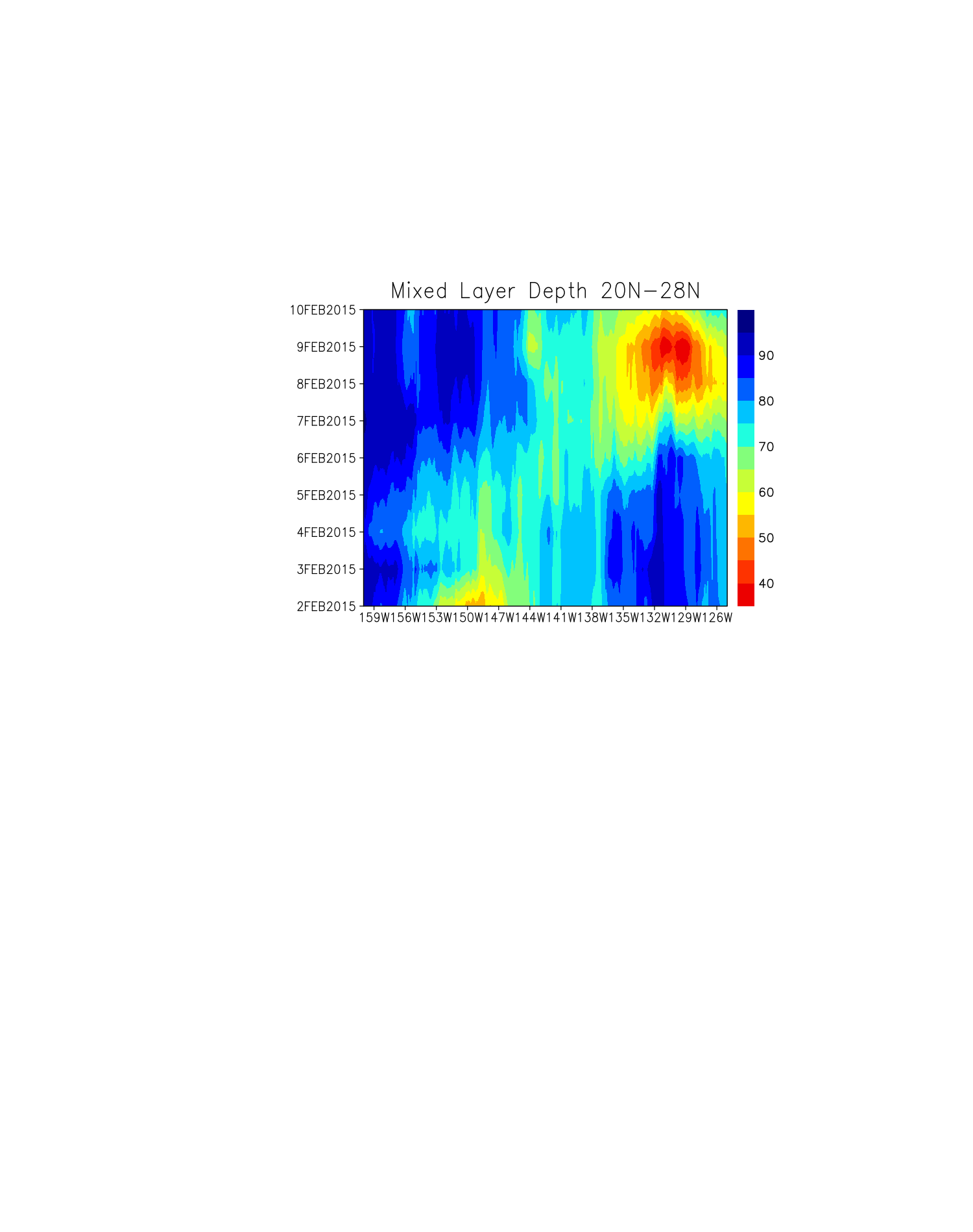


**Figure S4.** MLD averaged over 20ºN-28ºN during the AR event in early February, 2015 from the HYCOM reanalysis.


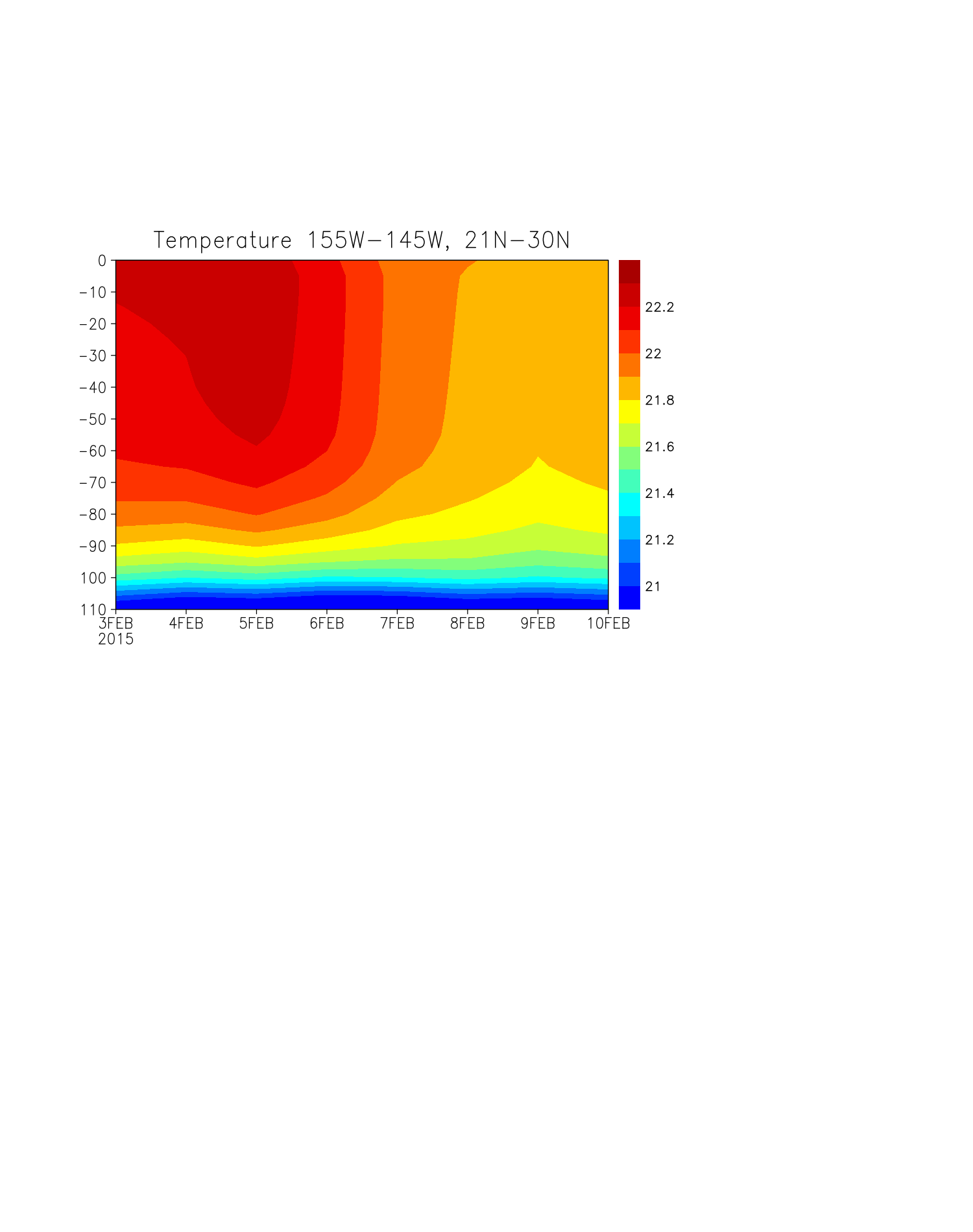


**Figure S5.** Time series of average temperature for the area 155ºW-145ºW, 21ºN-30ºN from the HYCOM reanalysis.


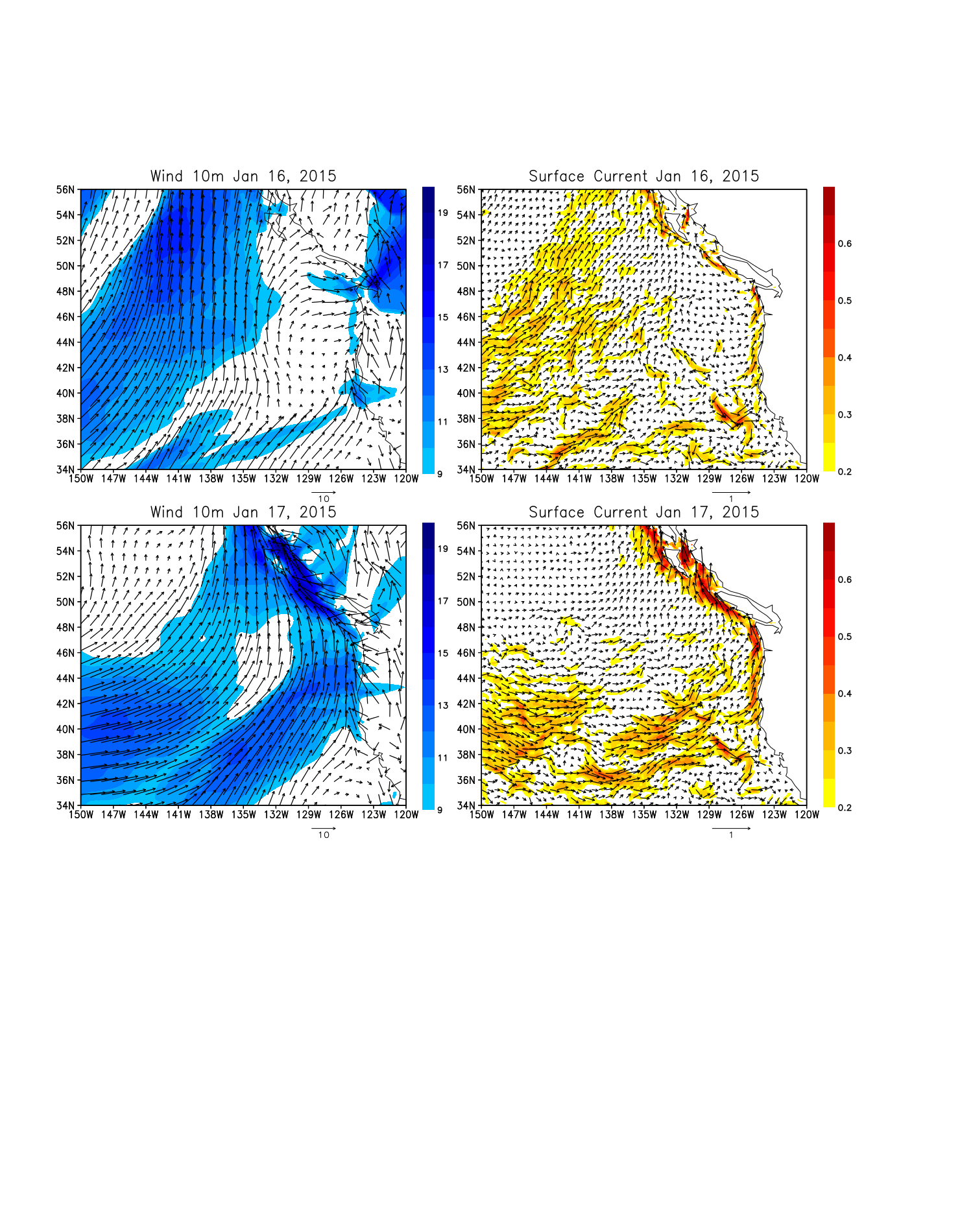


**Figure S6**. Left panels: Winds at 10 m height on January 16 (upper panel) and January 17 (lower panel), 2015 from the CFSV2 reanalysis. Shading indicates wind speed (m/s). Right panels: Surface currents on January 16 (upper panel) and January 17 (lower panel), 2015 from the HYCOM reanalysis. Shading indicates current speed (m/s).


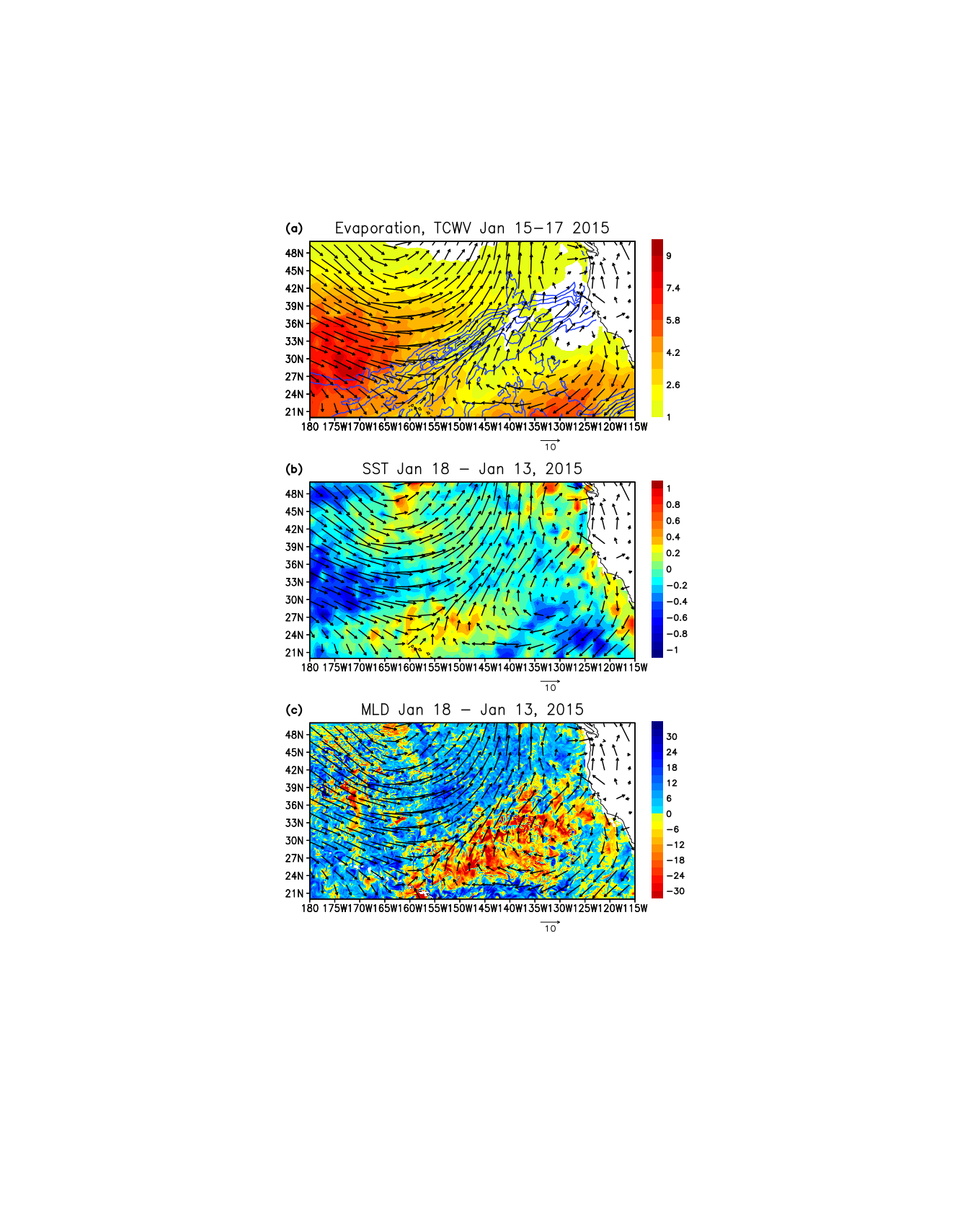


**Figure S7.**  . (a) Evaporation (mm/day: shading) on January 15-17, 2015, winds (m/s) at 10 m (arrows) on January 16, and total column integrated water vapor (contour) on January 16. The contour starts from 20 mm and the interval is 4 mm. (b) The difference of SST (°C) from OAFlux between the periods before the AR event (January 12-14, 2015) and after the event (January 17-19, 2015), and winds (m/s) at 10 m (arrows) on January 16, 2015. (c) Same as (b) except for the mixed layer depth (m) from the HYCOM reanalysis.


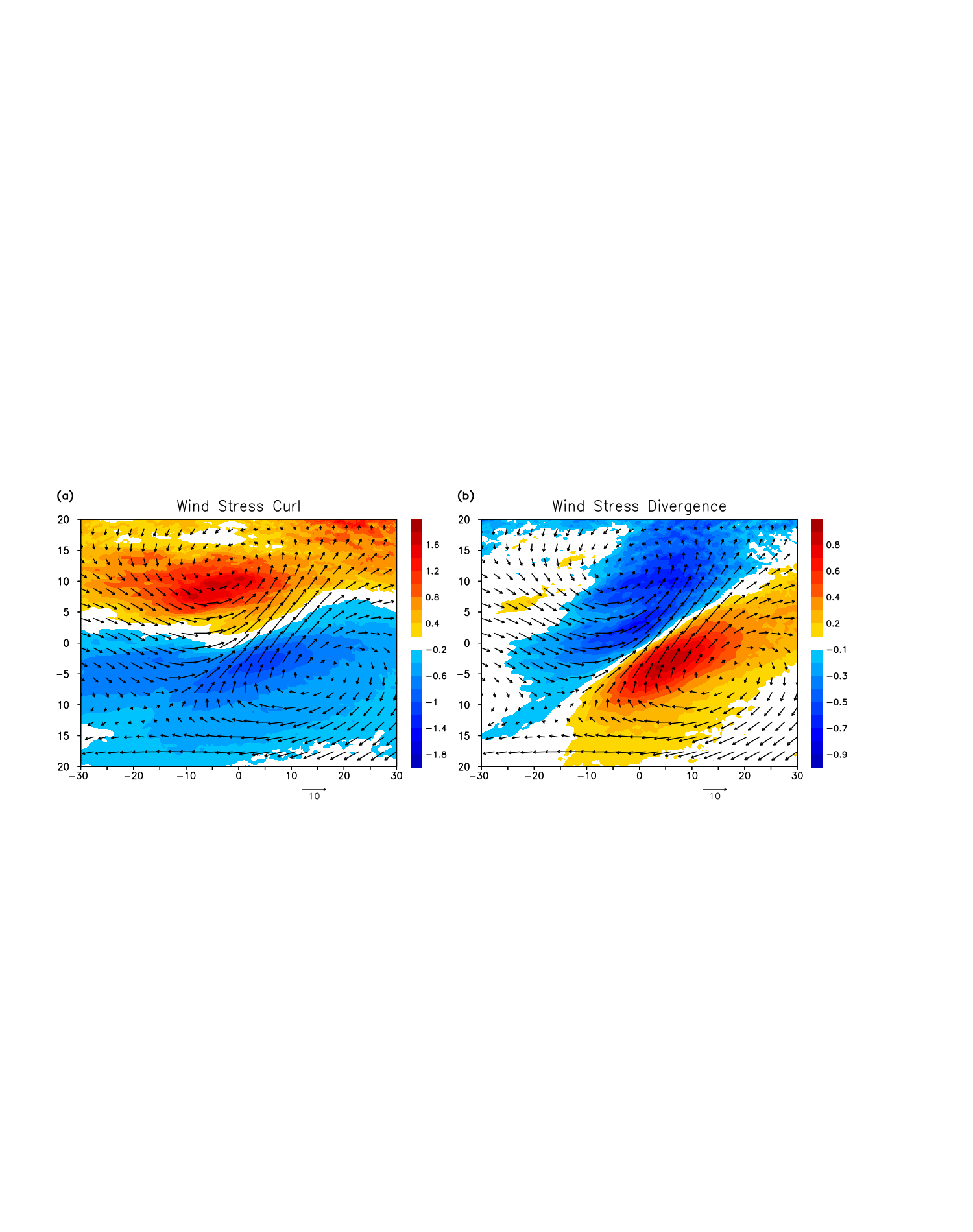


**Figure S8**. (a) Composite of wind stress curl (10^-6^ N m^-3^; shading) and surface winds (arrows) from the CFSV2 reanalysis (b) Composite of wind stress divergence (10^-6^ N m^-3^; shading) and surface winds (arrows) from the CFSV2 reanalysis.
